# Supplementary material for: Chromothripsis during telomere crisis is independent of NHEJ, and consistent with a replicative origin
Source: Genome Res. 2019 May;29(5):737–49. doi: 10.1101/gr.240705.118 (PMC6499312; doi:10.1101/gr.240705.118)
Supplement: Supplemental Material [file supp_gr.240705.118_Supplemental_file_1.zip › contigs/annotated_contigs/DB51/contig.3.DB51_length_443_mean_cov_4.57110609481.docx]

**DB51_length_443_mean_cov_4.57110609481**

```
ATTGATTATAAGACCAAATCCTCGAACATCTTAAAACATTTTTACATTGTAAATTAGATGGGAAAAAAAACTGAAAATTAAGCTTTGTG
 >chr1:72407913-72408154 + E=2e-124 p=0e+00
AATTTTTCTTACTTTTTTCATGCAGAACTATACTTAACGCCTCTCTGAAATCTGTGAAGTTTAATGTAATCAAGTGATGGTCTTAATTC

ATATAATTTAGTTTTACTTTAATGCACAAATTAAAATTCTTTAAATCTATATATATATATATA|GCT|AAGCTAAGTTTATATATATAT
                                                                   >chr3:77041488-77041
ATATATATATATATATATATA|G|CAAGCTAAGTTTTTCTTGGCAAATAAACTTTTCACACTTTCAAGGATGTCACTGTCCATTGTAGT
530 - E=1e-10 p=1e-03  >chr1:72517158-72517314 - E=2e-82
TTTCAAACTGGTCCCAGGAAATAAGAAATTTTTTTCGATGCAACAAAATAATATCGCATAATAGAAATGACAACAATTTTGTAGATATA

GT
```
